# Isogenic Transplantation of Hybrid Artificial Pleural Tissue Consisting of Rat Cells and Polyglycolic Acid Nanofiber Sheet Induces Restoration of Mesothelial Defects in Rat Model

**DOI:** 10.1111/aor.14947

**Published:** 2025-01-16

**Authors:** Kengo Tani, Daisuke Kimura, Yoshiya Asano, Cheng‐Yang Song, Hiroshi Shimoda, Masahito Minakawa

**Affiliations:** ^1^ Department of Thoracic and Cardiovascular Surgery Hirosaki University Graduate School of Medicine Hirosaki Aomori Japan; ^2^ Department of Neuroanatomy, Cell Biology and Histology Hirosaki University Graduate School of Medicine Hirosaki Aomori Japan; ^3^ Department of Thoracic Surgery Fourth Affiliated Hospital of China Medical University Shenyang Liaoning China; ^4^ Department of Anatomical Science Hirosaki University Graduate School of Medicine Hirosaki Aomori Japan

**Keywords:** artificial pleural tissue, cell‐accumulation method, mesothelial cells, polyglycolic acid nanofiber sheet, tissue engineering

## Abstract

**Background:**

Impairment of the visceral pleura following thoracic surgery often leads to air leaks and intrathoracic adhesions. For preventing such complications, mesothelial cell proliferation at the pleural defects can be effective. To develop new materials for pleural defects restoration, we constructed a hybrid artificial pleural tissue (H‐APLT) combining polyglycolic acid (PGA) nanofiber sheets with a three‐dimensional culture of mesothelial cells and fibroblasts and evaluated its therapeutic efficacy in a rat pleural defect model.

**Methods:**

After rat lungs were harvested, pleural mesothelial cells and lung fibroblasts were cultured separately. To construct H‐APLT, the cells were then coated with multiple layers of fibronectin and gelatin, followed by a single layer of mesothelial cells on top of multiple layers of fibroblasts accumulated onto a collagen‐coated PGA nanofiber sheet. Left lateral thoracotomy was performed, and H‐APLTs were transplanted into a rat model with pleural defects (*N* = 8). After 2–12 weeks of transplantation, lung resection and histological analyses were performed.

**Results:**

H‐APLTs exhibited a pleural structure with a highly integrated mesothelial layer in vitro. After transplantation, all eight rats survived until sacrifice. At 12 weeks post‐transplantation, the mesothelial layer on the lung surface was observed to be without defects with no intrathoracic adhesions detected.

**Conclusion:**

Successful isogenic engraftment of H‐APLTs was achieved in a rat model of pleural defects. The combination of accumulated fibroblasts and collagen‐coated PGA nanofiber sheets contributed to the maintenance of the mesothelial layer's structure and function, potentially preventing air leaks and intrathoracic adhesions.

## Graphical abstract

We have successfully constructed hybrid 3D tissue using mesothelial cells, fibronectin‐gelatin nanofilm‐coated fibroblasts, and collagen‐coated PGA nanofiber sheets to create artificial pleura, and achieved successful isogenic engraftment in a rat model of pleural defects.

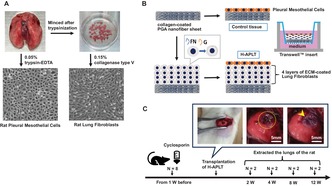

## 1 Background

The pleura consists of the mesothelium and connective tissue lining on the pleural cavity and surface of the lung [1]. The pleural cavity is filled with a minimal amount of serous fluid containing immunoglobulins, complements, lysozymes, and other proteins that eliminate microorganisms [2]. The mesothelium, a single layer of mesothelial cells, covers the luminal side of the parietal and visceral pleura. The pleural mesothelium serves as a semipermeable membrane and is constantly exposed to serous fluid, which helps regulate fluid and cellular transport [1, 2]. Mesothelial cells create a smooth, non‐adhesive surface that supports lung parenchyma movement by secreting glycocalyx [2, 3]. Additionally, these cells play a role in various defense mechanisms such as inflammatory and immune responses, tissue repair, coagulation, and fibrinolysis [1, 2, 3, 4].

Visceral pleural defects, occasionally caused by thoracic surgery, result in prolonged air leakage from the lung parenchyma. Impaired or delayed mesothelial cell repair leads to intrathoracic adhesion and fibrosis [1, 2, 3, 4]. Long‐term air leaks prolong chest drainage duration, restrict patient mobility, cause pain, and increase the risk of intrathoracic infections [5, 6]. Preventing air leaks is effective in preserving respiratory function after lung resection [7]. Moreover, intrathoracic adhesions inhibit respiratory lung motion and exacerbate restrictive ventilation disorders [8]. Pleural defects can be treated by suturing, stapling, and electrocautery. However, they may rather aggravate pulmonary air leaks in the fragile lung parenchyma or pleura, such as emphysema or interstitial pneumonia [9].

Although fibrin glue is utilized as an alternative, its bonding strength remains weak [10, 11]. Therefore, the combination of polyglycolic acid (PGA) sheets and fibrin glue has been utilized by several research groups [7, 8, 9, 10, 11, 12, 13]. The PGA sheet prevents the removal of glue and ensures the secure formation of fibrin between the pleural defect and sheet [9, 12]. It also induces biological tissue regeneration by surrounding connective tissues infiltrating between PGA fibers, concomitant with biodegradation of the fibers. However, PGA triggers a severe and prolonged inflammatory reaction due to acidification [13]. Thus, it is problematic as it causes intrathoracic adhesion. Recently, PGA sheets with finer fibers (PGA nanofiber sheets) were developed, enabling a reduction in inflammatory reactions due to residual fibers [13, 14]. The areal density of the PGA nanofiber sheet (Neoveil nano, D‐5, 5.2 g/m^2^, Gunze Ltd., Kyoto, Japan) is approximately one‐sixth that of the conventional PGA sheet (Neoveil, 015G, 33.9 g/m^2^, Gunze Ltd.). As a result, the nanofiber sheet is expected to decompose quickly and cause less tissue reaction [13]. PGA sheets undergo non‐enzymatic hydrolysis, producing glycolic and lactic acids, which can trigger inflammation due to the resulting acidification. These acids are then metabolized into water and carbon dioxide over a period of 15 weeks [13]. In contrast, PGA nanofibers are 93% hydrolyzed in 4 weeks in vitro, according to Gunze Ltd. The tensile strength of the PGA nanofiber sheet (Neoveil nano, D‐5, 0.05 mm thickness) is 2.64 MPa, while that of the conventional PGA sheet (Neoveil, 015G, 0.15 mm thickness) is 6.97 MPa. Thinner PGA nanofiber sheets have a higher affinity for lung parenchyma than conventional sheets, as tensile strength decreases with thinning and tissue attachment improves. However, even when PGA nanofiber sheets are used, intrathoracic adhesion remains a concern. To address this issue, we considered that promoting mesothelial cell formation at the pleural defect is essential.

Transplantation of artificial pleural tissues constructed from cells is an ideal treatment for lung air leaks that prevents lung adhesion. The development of artificial pleural tissues using the cell sheet technique has been reported with successful transplantation [15, 16, 17, 18, 19, 20]. However, in therapeutic applications for human lungs, sufficient tissue thickness and mechanical strength must be maintained for the long term. Most tissue cells in the body are embedded within the fibrous extracellular matrix (ECM), which is generally composed of fibronectin and collagen [21]. To mimic actual biological tissues, it is more effective to implant three‐dimensional (3D), multilayered cells rather than a single cell layer [21, 22]. Therefore, artificial 3D tissue can be a promising material for pleural regenerative medicine.

In the early stages of cell culture, the amount of ECM secreted on the cell surface is insufficient [21]. To develop 3D artificial tissue in a simple process, we have established a tissue construction technique known as the “cell accumulation method” in previous studies [23, 24]. In this technique, cell cultures are coated by “ECM nanofilm” with multiple layers of fibronectin and gelatin [21, 22, 23, 24, 25]. The properties of fibronectin‐gelatin (FN‐G) nanofilms are as follows. Fibronectin is a multifunctional glycoprotein that interacts with various ECM proteins. It interacts with α1 chain of collagen via a gelatin‐binding domain, and binds to α5β1 integrin, which are cell adhesion molecules on cell membranes, via Arg‐Gly‐Asp (RGD) motifs [26, 27]. The interaction between α5β1 integrin and fibronectin is essential for cell attachment, migration, proliferation, and differentiation [21]. In the cell accumulation method, the FN‐G nanofilms applied to individual cell surfaces by laminating several layers of fibronectin and gelatin anchored by the α5β1 integrin on the cell membrane, providing uniform cell–cell adhesion among all cells seeded in a 3D accumulation [21, 25]. Through this method, an artificial human peritoneal tissue consisting of layered fibroblasts with vessels and mesothelial cells was developed [24]. In this tissue, mesothelial cells form a highly integrated sheet mimicking the mesothelium with intercellular junctions, microvilli, and a basement membrane [24].

The combination of 3D artificial pleural tissue and PGA nanofiber sheets can generate a biomaterial for lung regenerative medicine with a highly integrated mesothelium and a firm and biodegradable scaffold. Therefore, this study aimed to develop a hybrid artificial pleural tissue (H‐APLT) by fusing the cell accumulation method using rat lung fibroblasts, mesothelial cells, and collagen‐coated PGA nanofiber sheets. Furthermore, isogenic transplantation of H‐APLTs was performed in a rat model with mesothelial defect induction and histologically evaluated tissue engraftment and pleural repair with the mesothelium.

## 2 Materials and Methods

### 2.1 Animals and Anesthetics

All procedures were authorized by the Animal Research Committee of Hirosaki University and were carried out in accordance with the institution's guidelines for animal experimentation. This study utilized 10‐week‐old female Wistar rats (CLEA Japan Inc., Tokyo, Japan), which were maintained under controlled light (12‐h light: 12‐h dark cycle) and a temperature of 21°C.

Rats were anesthetized with an intraperitoneal injection of medetomidine hydrochloride (0.75 mg/kg, Kyoritsu Seiyaku Co., Tokyo, Japan), midazolam (4.0 mg/kg, Sandoz, Tokyo, Japan), and butorphanol (5.0 mg/kg, Meiji Animal Health Co. Ltd., Kumamoto, Japan). Atipamezole (1.5 mg/kg, Kyoritsu Seiyaku Co.) was administered as an antagonist.

For immunosuppression, cyclosporin (Neoral, Novartis, Rueil‐Malmaison, France) was added to drinking water at a concentration of 100 mg/L, starting 1 week prior to and continuing throughout the transplantation period.

### 2.2 Reagents

Dulbecco's modified Eagle's medium (DMEM; low glucose) and alpha modified Eagle's medium (α‐MEM) were obtained from Sigma‐Aldrich (St. Louis, MO, USA) and Nacalai Tesque (Kyoto, Japan), respectively. All media were supplemented with 10% fetal bovine serum (FBS; Nichirei Biosciences Inc., Tokyo, Japan), 1% penicillin–streptomycin (Wako Pure Chemical Industries Ltd., Osaka, Japan) and 2.5 μg/mL amphotericin B (Wako Pure Chemical Industries Ltd.). For cell isolation and culture, 0.05% trypsin–EDTA (Wako Pure Chemical Industries Ltd.), 0.15% collagenase type V (Wako Pure Chemical Industries Ltd.), and 100 mm diameter culture dishes (Thermo Fisher Scientific Inc. Waltham, MA, USA) were utilized. Bovine plasma‐derived fibronectin and porcine‐skin‐derived gelatin were purchased from MP Biomedicals Inc. (Santa Ana, CA, USA) and Sigma‐Aldrich, respectively. Porcine‐tendon‐derived collagen solution (Cellmartix Type I‐A) was obtained from Nitta Gelatin Inc. (Osaka, Japan). Mouse anti‐podoplanin monoclonal antibody was acquired from Novus Biologicals LLC (Centennial, CO, USA). Mouse anti‐CD31 monoclonal antibody was obtained from Becton, Dickinson and Company (Franklin Lakes, NJ, USA) and rabbit anti‐vimentin polyclonal antibody was purchased from Proteintech Group Inc. (Rosemont, IL, USA). Alexa Fluor 594‐conjugated goat anti‐mouse IgG and Alexa Fluor 488‐conjugated goat anti‐rabbit IgG were sourced from Thermo Fisher Scientific Inc.

### 2.3 Isolation and Culture of Pleural Mesothelial Cells and Lung Fibroblasts

**FIGURE 1:**
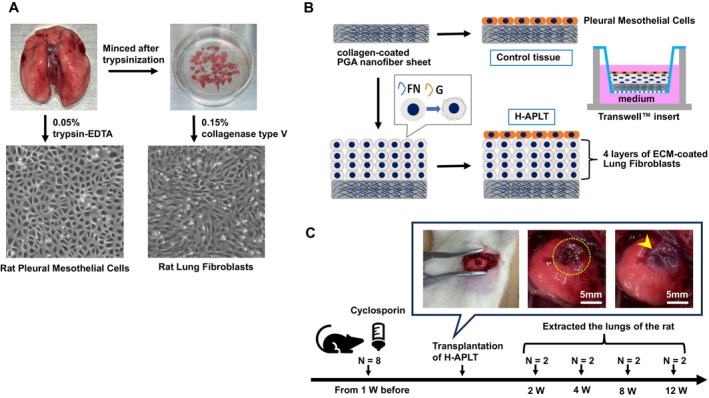
Construction of H‐APLT and transplantation of H‐APLT into a rat lung air leak model. (A) Isolation and culture of pleural mesothelial cells and lung fibroblasts. Rat lungs were treated with 0.05% trypsin–EDTA, and pleural mesothelial cells were isolated. Lungs were then minced and treated with 0.15% collagenase type V, the lung fibroblasts were isolated. Pleural mesothelial cells were characterized as having a cobblestone‐like arrangement, whereas fibroblasts were spindle‐shaped. (B) Construction of artificial tissues by cell accumulation technique. Lung fibroblasts with fibronectin‐gelatin nanofilms accumulated on the collagen‐coated PGA nanofiber sheet. A 3D tissue comprising four layers of lung fibroblasts was formed. Pleural mesothelial cells were cultured on top of these layers to generate H‐APLTs. The cells were cultured in Transwell inserts to create H‐APLT. The control tissue consisted of a single layer of pleural mesothelial cells on a collagen‐coated PGA nanofiber sheet. (C) The timeframe for implanting the H‐APLT into the rat lung air leak models. For immunosuppression, cyclosporin was added to drinking water (100 mg/L), 1 week before and throughout the transplantation period. Left lateral thoracotomy was performed. An air leak was generated via cutting the lung parenchyma into an approximately 5 mm long incision with a depth of 3 mm using surgical scissors (yellow dotted line). H‐APLTs (6 mm in diameter, yellow arrowhead) were transplanted into the air‐leak site. A collagen solution was instilled over the H‐APLT to prevent dislodgement. At 2, 4, 8, and 12 weeks post‐transplantation, the rats were euthanized. At each time point post transplantation, two rats were assessed (total *N* = 8). ECM, extracellular matrix; FN, fibronectin; G, gelatin; H‐APLT, hybrid artificial pleural tissue; PGA, polyglycolic acid. [Color figure can be viewed at wileyonlinelibrary.com]

Pleural mesothelial cells and fibroblasts were isolated and cultured from rat lungs according to previous studies [16, 28]. Lung was harvested from rats, washed three times with phosphate‐buffered saline (PBS), and incubated in 10 mL of 0.05% trypsin–EDTA for 40 min at 37°C. Lung surface was rubbed using a cell scraper. Cell suspensions were collected, and 30 mL of DMEM was added. The mixed cell suspension was centrifuged at 180 × *g* for 5 min. Pleural mesothelial cells were resuspended in DMEM and seeded in 100‐mm diameter culture dishes. Lung fibroblasts were collected as follows: lung tissues, after harvesting pleural mesothelial cells, were minced into 3 × 3 mm pieces and treated with 0.15% collagenase type V for 90 min. The cell suspension was collected and centrifuged at 180 × *g* for 5 min. Cells were resuspended in α‐MEM and seeded on 100‐mm dishes as lung fibroblasts. Cells were cultured at 37°C with 5% CO_2_, and the culture medium was changed every other day. After 1 week, both pleural mesothelial cells and lung fibroblasts became confluent (Figure 1A).

### 2.4 Immunofluorescence Analysis of Pleural Mesothelial Cells and Lung Fibroblasts

Pleural mesothelial cells and lung fibroblasts were seeded onto Nunc Lab‐Tek Chamber Slide System (177402PK, 8‐well, Thermo Fisher Scientific Inc.) at a density of 1 × 10^4^ cells per well, respectively. After discarding the cell culture medium, the cells were fixed with 4% paraformaldehyde in 0.1 M phosphate buffer (pH 7.4) for 1 h at 4°C. The cells were then washed with PBS, soaked with 0.1 M glycine for 15 min at room temperature, and permeabilized with 0.25% Triton X‐100 in PBS for 30 min at room temperature. Subsequently, the cells were treated with 2% normal goat serum (Wako Pure Chemical Industries Ltd.) in PBS with 0.05% Tween 20 for 1 h at room temperature. They were then stained with primary antibodies (1:200 dilution) for double staining of podoplanin/vimentin and CD31/vimentin overnight at 4°C. Next, immunoreactions were visualized using fluorescence‐labeled secondary antibodies (1:200 dilution). The cell nuclei were stained with 4′,6‐diamidino‐2‐phenylindole (DAPI). Specimens were observed under a fluorescence microscope (BZ‐X700, Keyence, Tokyo, Japan).

### 2.5 Construction of the H‐APLT

PGA nanofiber sheet (Neoveil nano, D‐5, Gunze Ltd.) was cut into circular sheets with 6 mm in diameter and sterilized with UV light. These circular sheets were then placed onto Transwell inserts with porous polyester bottom for 24‐well culture plate (3470, pore size: 0.4 μm, Corning, New York, USA) and coated with porcine tendon‐derived collagen solution (Cellmartix Type I‐A) for 10 min at 37°C [29, 30, 31]. Using this setup, a multi‐layered 3D tissue was constructed using the cell accumulation method with the layer‐by‐layer cell coating technique, as previously reported [21, 23, 24, 25]. Briefly, cultured lung fibroblasts were collected via trypsinization within passage one. Fibroblasts were suspended in 0.04 mg/mL fibronectin in 50 mM Tris–HCl buffer (pH 7.4) and incubated for 1 min with gentle rotation. After washing with 50 mM Tris–HCl buffer, fibroblasts were treated with 0.04 mg/mL gelatin in 50 mM Tris–HCl buffer. This process of alternating fibronectin and gelatin treatments for nine steps resulted in an approximately 10‐nm‐thick coating of the FN‐G nano‐film on each fibroblast. Coated cells were suspended in DMEM and seeded onto Transwell inserts at a density of 4 × 10^5^ cells per well. The inserts were placed in 24‐well cell culture plates containing similar medium. The following day, after aspirating the medium, 5 × 10^4^ pleural mesothelial cells per well were seeded on top of the lung fibroblast layer. Consequently, the equivalent of four layers of lung fibroblasts (based on previous studies) [23, 24] and a single layer of pleural mesothelial cells were applied to the collagen‐coated PGA nanofiber sheet for constructing H‐APLTs (Figure 1B). The tissues were then cultured in DMEM at 37°C with 5% CO_2_ for 5 days, with medium changing every other day. Control tissues, consisting of only a single layer of pleural mesothelial cells on the collagen‐coated PGA nanofiber sheets without lung fibroblasts, were also constructed (Figure 1B).

### 2.6 Histological Evaluation of the Artificial Tissues

The H‐APLTs and control tissues were fixed with either 4% paraformaldehyde in 0.1 M phosphate buffer (pH 7.4) for light microscopy or with 2% paraformaldehyde and 2.5% glutaraldehyde in 0.1 M phosphate buffer (pH 7.4) for scanning electron microscopy (SEM). For light microscopy, the fixed tissues were embedded in paraffin, and 5‐μm‐thick sections were stained with hematoxylin and eosin (H&E). For immunohistochemistry, antigen retrieval was performed by boiling the deparaffinized sections twice in 10 mM citric acid (pH 6.0) in a microwave oven at 500 W for 5 min each. The sections were then treated with 3% normal goat serum (Wako Pure Chemical Industries Ltd.) in 0.1 M phosphate buffer (pH 7.4) containing 0.05% Triton X‐100 for 1 h at room temperature and incubated with primary antibodies (1:100 dilution) for double staining of podoplanin and vimentin for two nights at 4°C. Immunoreactions were then visualized using fluorescence‐labeled secondary antibodies (1:200 dilution) and specimens were observed under a fluorescence microscope (BZ‐X700, Keyence).

For SEM analysis, the fixed tissues were first treated with 1% tannic acid in 0.1 M phosphate buffer (pH 7.4), followed by post‐fixation in 1% osmium tetroxide in the same buffer. The tissues were then dehydrated with ethanol, transitioned to t‐butyl alcohol, and frozen at −20°C. After the t‐butyl alcohol was removed by freeze‐drying, the tissues were coated with a 100 Å thick layer of platinum and examined using a scanning electron microscope (JSM‐6510LV, JEOL, Tokyo, Japan).

### 2.7 Transplantation of H‐APLT Into a Rat Lung Air Leak Model

Rats were anesthetized with an intraperitoneal injection of a combined triadic anesthesia (medetomidine hydrochloride, midazolam, and butorphanol). Intratracheal intubation was performed using the outer tube of a 16‐gauge needle (Terumo Co. Ltd. Tokyo, Japan). The rats were then ventilated with air at a rate of 60 breaths per minute and a tidal volume of 0.01 mL/g. A left lateral thoracotomy was performed in the fifth intercostal space (Figure 1C). A visceral pleural defect was created by making an incision approximately 5 mm long and 3 mm deep in the lung parenchyma using surgical scissors and the presence of an air leak was confirmed visually. Hemostasis was then achieved by applying compression with a cotton swab. H‐APLTs were transplanted onto the air leak site (Figure 1C). Then 50 μL of porcine‐tendon‐derived collagen solution (Cellmartix Type I‐A) was added over the H‐APLT. This collagen solution was applied for immobilization of H‐APLT until its engraftment due to the gelatinizing property at 37°C, around rat's body temperature. After chest closure, atipamezole was administered intraperitoneally, and the rats were extubated upon restoring spontaneous breathing.

### 2.8 Histological Examination of the Transplantation Site

At 2, 4, 8, and 12 weeks post‐transplantation, the rats were euthanized, and left lateral thoracotomy was performed in the fifth intercostal space. At each time point post transplantation, two rats were assessed (total *N* = 8). The left lung, including the transplanted site, was resected and fixed with 4% paraformaldehyde in 0.1 M phosphate buffer (pH 7.4) for light microscopy. The fixed tissues were embedded in paraffin, and 5‐μm‐thick tissue sections were prepared on glass slides. H&E staining was conducted for conventional tissue observation. For immunohistochemistry, antigen retrieval was performed by boiling the deparaffinized sections twice in 10 mM citric acid (pH 6.0) in a microwave oven at 500 W for 5 min each. Sections were then treated with 3% normal goat serum (Wako Pure Chemical Industries Ltd.) in 0.1 M phosphate buffer (pH 7.4) containing 0.05% Triton X‐100 for 1 h at room temperature and incubated with the primary antibody (podoplanin, 1:100 dilution) for two nights at 4°C. Immunoreactions were visualized using fluorescence‐labeled secondary antibodies (1:200 dilution). Specimens were observed under a fluorescence microscope (BZ‐X700, Keyence).

## 3 Results

### 3.1 Evaluation of Isolated Cells From Rat Lungs

**FIGURE 2:**
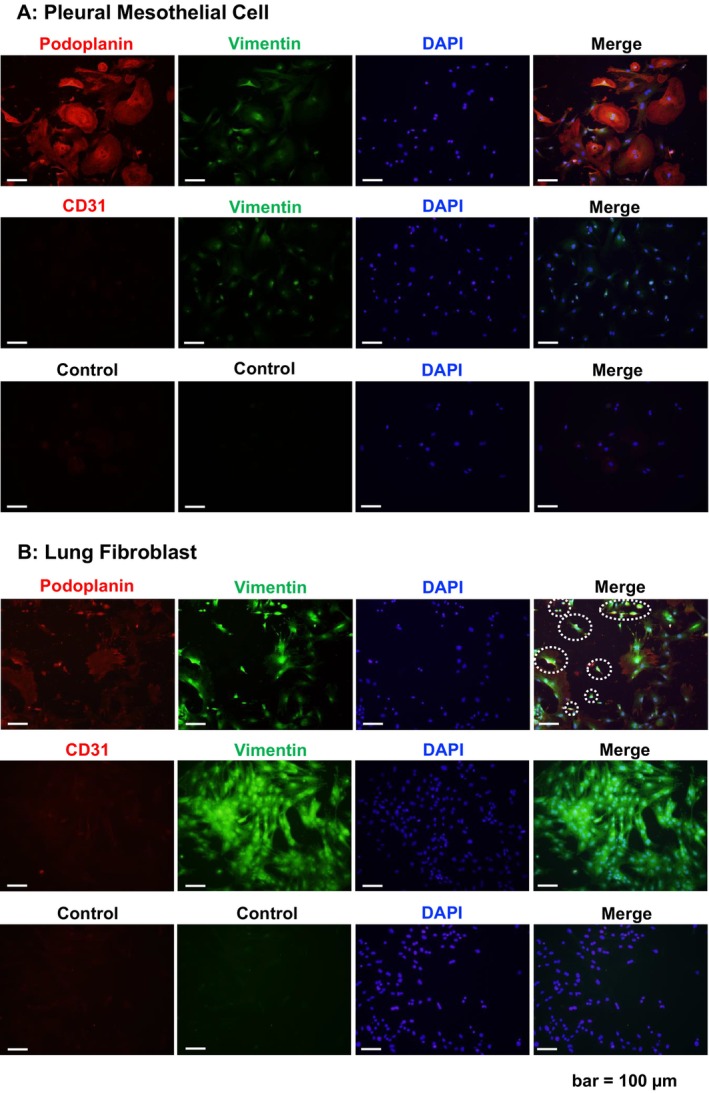
Immunofluorescence analysis of pleural mesothelial cells and lung fibroblasts. (A) Immunostaining findings of pleural mesothelial cells. Podoplanin was strongly positive and vimentin was weakly positive (upper panel). CD31 was negative (middle panel). The negative control without primary antibodies is shown in the lower panel. (B) Immunostaining findings of lung fibroblasts. Vimentin was strongly positive (upper and middle panels). CD31 was negative (middle panel). Some cells were weakly positive for podoplanin (white dotted line in the upper panel); these were thought to be pleural mesothelial cells. These staining results likely occurred because lung fibroblasts were collected after pleural mesothelial cells from the same lung. The negative control without primary antibodies is shown in the lower panel. [Color figure can be viewed at wileyonlinelibrary.com]

Immunofluorescence staining of isolated cells is shown in Figure 2. In Figure 2A, podoplanin was strongly positive, supporting the identification of mesothelial cells. The intermediate filament protein vimentin was weakly positive, which is consistent with its expected role as part of the mesothelial cell cytoskeleton. The vascular endothelial marker CD31 was negative. In Figure 2B, the strong positivity for vimentin and the negativity for CD31 confirmed the cells as fibroblasts. Some cells were positive for podoplanin (indicated by the dotted line). Although the mixture of pleural mesothelial cells was found in lung fibroblasts, it was proven that two types of cells were isolated and cultured from the same lung.

### 3.2 Evaluation of Tissue Structure and Mesothelial Integration in H‐APLT

**FIGURE 3:**
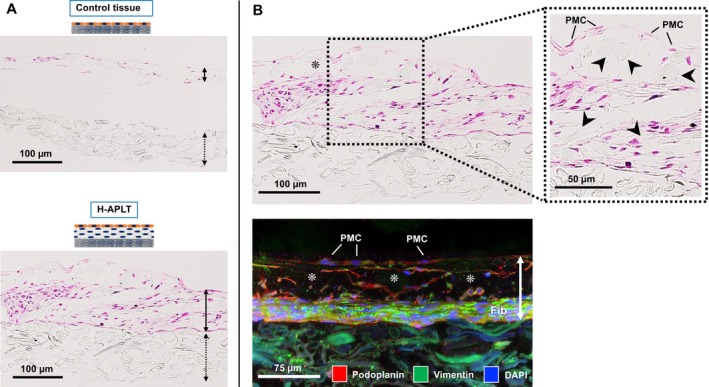
Construct findings of artificial tissue. (A) Hematoxylin and eosin staining of artificial tissues. The H‐APLT and the control tissue was observed 5 days after construction. Solid and dotted lines represent cells and PGA nanofiber sheets, respectively. In the control tissue, the cells detached from the PGA nanofiber sheet (upper panel). H‐APLT had a thicker cellular component, without separation between the cellular components and collagen‐coated PGA nanofiber sheet (lower panel). (B) In the H‐APLT, PGA nanofibers were identified within the cellular components (upper panel, arrowheads), demonstrating the integration of tissue structure with PGA nanofiber. Immunofluorescence staining of H‐APLT is shown in the lower panel. The solid line outlines the cellular components. Podoplanin‐positive cells were observed on the surface of H‐APLT, indicating the presence of pleural mesothelial cells (PMC). Numerous vimentin‐positive cells were observed on the substrate PGA nanofiber, suggesting the presence of lung fibroblasts (Fib). Podoplanin‐positive cells were also detected within the lung fibroblast layer, indicating a mixture of pleural mesothelial cells. These staining results likely occurred because lung fibroblasts were collected after pleural mesothelial cells from the same lung. The gaps between mesothelial cells and the fibroblast layer are considered artifactual spaces related to the preparation process for histological sections (asterisk). H‐APLT, hybrid artificial pleural tissue; PGA, polyglycolic acid. [Color figure can be viewed at wileyonlinelibrary.com]

Cross‐sections of artificial tissues are illustrated in Figure 3. For the control tissue, the mesothelial cell layer was detached from the PGA nanofiber sheet (Figure 3A upper panel), whereas H‐APLT had a thicker cellular component without separation between the cellular components and collagen‐coated PGA nanofibers (Figure 3A lower panel). Fibers were identified within the cellular components (Figure 3B upper panel, arrowheads) demonstrating fusion of the tissue structure and PGA nanofiber. Therefore, the tissue components were more integrated with the collagen‐coated PGA nanofiber sheet than with the control tissue.

Immunofluorescence staining of H‐APLT is shown in Figure 3B lower panel. Podoplanin‐positive cells detected on the H‐APLT surface suggest the presence of pleural mesothelial cells. Numerous vimentin‐positive cells were observed on the substrate PGA nanofiber, suggesting the presence of lung fibroblasts. Podoplanin‐positive cells were also observed, suggesting a mixture of pleural mesothelial cells. These cell staining results likely occurred because lung fibroblasts were collected after pleural mesothelial cells from the same lung.

**FIGURE 4:**
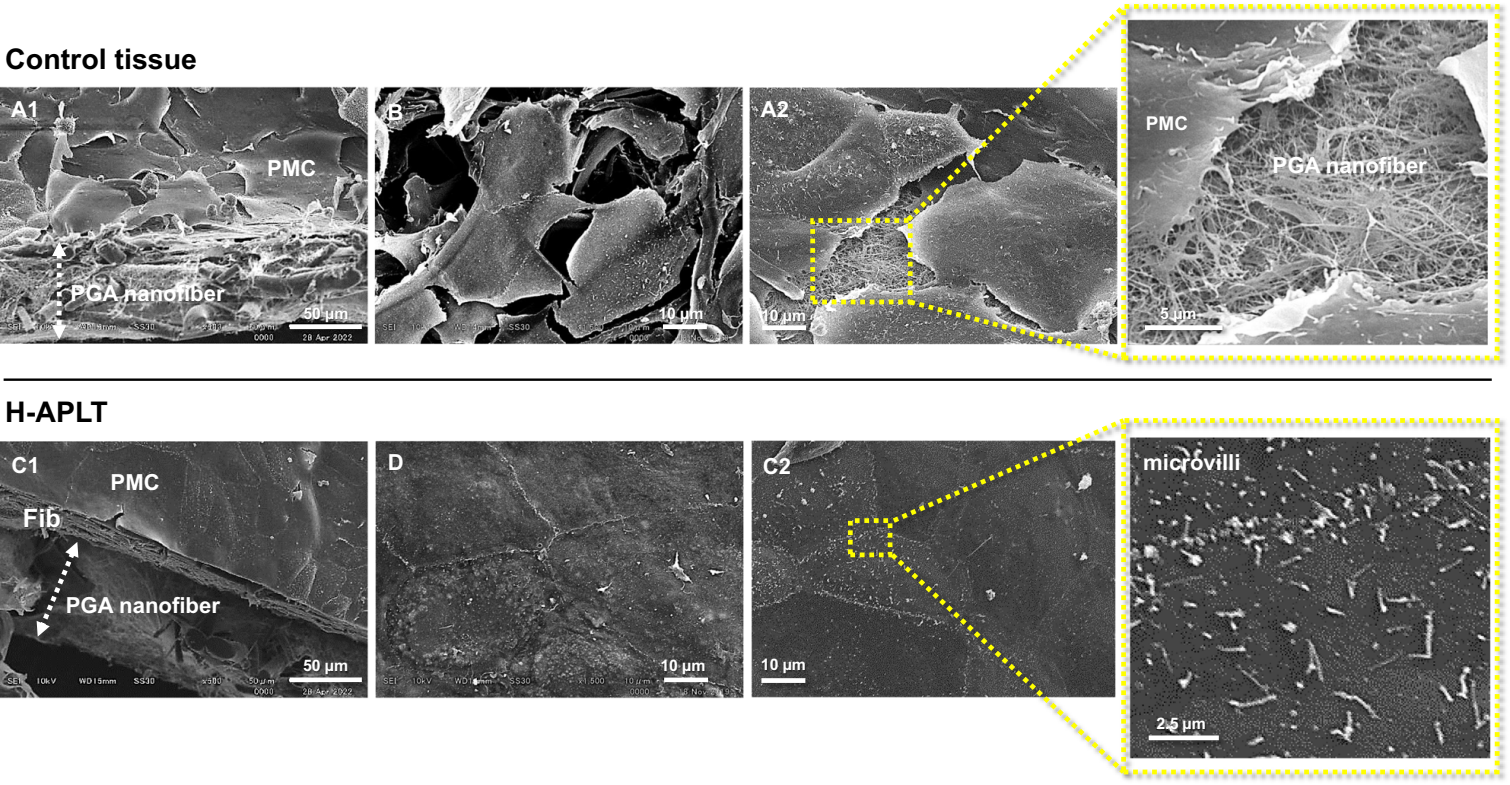
Scanning electron microscopy findings. (A, B) Are control tissues from different specimens. (C, D) are H‐APLT from different specimens. (A1) Uncomplete sheet structures. (A2, B) In the control tissue, the pleural mesothelial cells were detached from each other. (C1) In the H‐APLT, pleural mesothelial cells and fibroblasts formed sheet‐like structures. (C2, D) Pleural mesothelial cells were arranged in a cobblestone‐like pattern with no gaps between them. Numerous microvilli were identified in the high‐power field. Reproducibility has been demonstrated, confirming that these findings are not artifacts related to the SEM preparation process. Fib, fibroblasts; H‐APLT, hybrid artificial pleural tissue; PGA, polyglycolic acid; PMC, pleural mesothelial cells. [Color figure can be viewed at wileyonlinelibrary.com]

SEM images are exhibited in Figure 4. In the control tissue, the topmost mesothelial cells detached from each other (Figure 4A2,B), resulting in an incomplete sheet structure (Figure 4A1). Meanwhile, in H‐APLTs, mesothelial cells and fibroblasts presented a highly integrated sheet‐like structure (Figure 4C1). Pleural mesothelial cells were arranged in a cobblestone‐like pattern, with no gaps between them (Figure 4C2,D). Numerous microvilli, which are features of normal mesothelium, were observed on the surface of the sheets (Figure 4C2 high magnification).

### 3.3 Transplantation of H‐APLT

**FIGURE 5:**
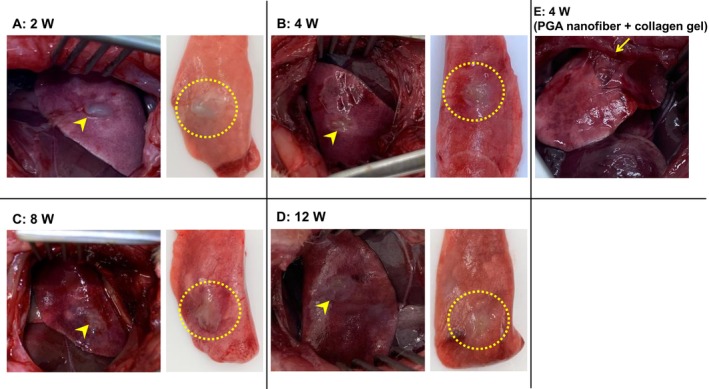
Macroscopic findings in the thoracic cavity and lungs after H‐APLT transplantation. H‐APLTs grew into the tissue, and the engrafted sites gradually became obscured (yellow arrowhead). The engrafted site is marked with a yellow dotted line. The H‐APLT was identified as a thick transplant at 2 weeks after surgery (A), but gradually integrated into the host tissue along the postoperative course (B–D). No intrathoracic adhesions were observed at any time point. However, 4 weeks after the attachment of a rat with only a PGA nanofiber sheet and collagen gel, adhesion between the parietal pleura and lung parenchyma was observed (E, yellow arrow). H‐APLT, hybrid artificial pleural tissue; PGA, polyglycolic acid. [Color figure can be viewed at wileyonlinelibrary.com]

All eight transplanted rats survived until they were sacrificed for tissue collection. Macroscopic findings of H‐APLT‐transplanted lungs at 2, 4, 8, and 12 weeks post‐surgery revealed no intrathoracic adhesions (Figure 5). H‐APLTs were engrafted and gradually fused into the host tissue, making the graft edge obscure over time (Figure 5). At 2 weeks, H‐APLTs remained thick and clearly identifiable (Figure 5A). At 4 weeks, they were integrated within the host tissue compared with those at 2 weeks (Figure 5B). The tissue further healed at 8 and 12 weeks (Figure 5C,D). When only a PGA nanofiber sheet and collagen gel were applied to the pleural defect, adhesion between the parietal pleura and lung parenchyma was observed (Figure 5E).

**FIGURE 6:**
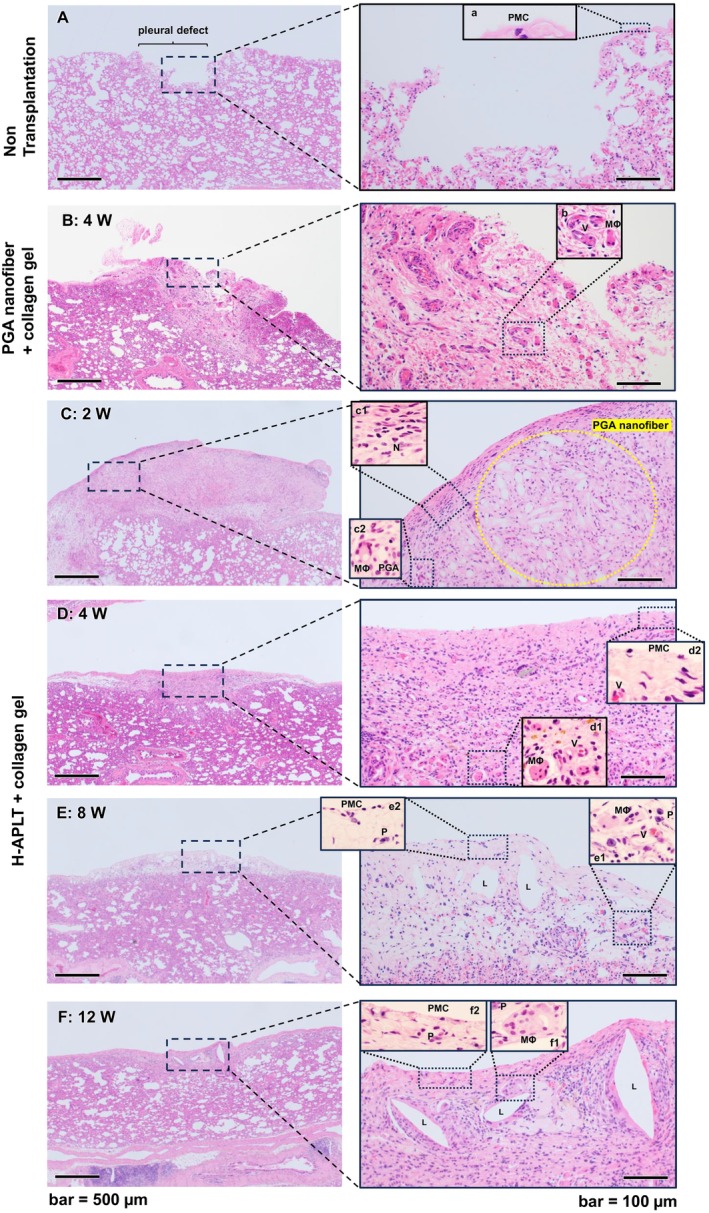
Microscopic findings of engrafted site of the H‐APLT (Hematoxylin–eosin staining). The right‐hand images are magnified images of the black dotted boxes in the left‐hand images. (A) A pleural defect model. The alveolar structures were exposed. Only a few pleural mesothelial cells are present (a). (B) Four weeks after the attachment of the PGA nanofiber sheet and collagen gel. Although no remnants of the PGA nanofiber were seen, numerous instances of neovascularization were observed, suggesting a strong inflammatory response. Multinucleated macrophage (MΦ) and neovascular vessels (V) are shown in (6b). (C) Two weeks post‐transplantation, engraftment of H‐APLT was confirmed. Remnants of the PGA nanofibers were observed with abundant surrounding connective tissues (yellow dotted line). Immune cells including neutrophils (c1) and multinucleated macrophages (c2) were also identified. (D) After 4 weeks, the pleural defect was thoroughly replaced by thinned transplanted tissue. The PGA nanofibers were completely degraded, although multinucleated macrophages were still present (c1). Neovascularization was observed (d1 and d2, V). The surface of the graft was covered by flattened pleural mesothelial cells with fibrous connective tissue (d2). (E) After 8 weeks, macrophages, neovascularization and plasma cells were observed (e1). Dilated luminal structure (L) developed in the connective tissue. Pleural mesothelial cells were identified on the surface (e2). (F) After 12 weeks, the transplanted tissue showed development of luminal structures (L) with stromal fibrosis. Multinucleated macrophages were observed around the luminal structures (f1). Pleural mesothelial cells were identified on the surface (f2). H‐APLT, hybrid artificial pleural tissue; L, luminal structure; MΦ, macrophage; P, plasma cell; PGA, polyglycolic acid; PMC, pleural mesothelial cell; V, neovascularization. [Color figure can be viewed at wileyonlinelibrary.com]

Microscopic observations of H‐APLT‐transplanted lungs are exhibited in Figure 6C–F, including histological images of the pleural defective lung (Figure 6A) and only attachment of PGA nanofiber sheet and collagen gel (Figure 6B). Four weeks after the attachment of the PGA nanofiber sheet and collagen gel, numerous instances of neovascularization were observed, suggesting a strong inflammatory response (Figure 6B).

At 2 weeks, the graft was observed as thickened tissue (Figure 6C left), and remnants of the PGA nanofibers were observed with abundant surrounding connective tissues (Figure 6C right, yellow dotted line). Immune cells including neutrophils (Figure 6c1) and multinucleated macrophages (Figure 6c2) were also identified. These cells are involved in the inflammation and biodegradation of PGA nanofibers.

At 4 weeks, the transplanted site with the graft had thinned (Figure 6D left), and the pleural defect appeared to be replaced with fibrous connective tissue (Figure 6D right). Additionally, the PGA nanofibers completely disappeared, although macrophages were still present (Figure 6d1), suggesting the continuation of biodegradation.

**FIGURE 7:**
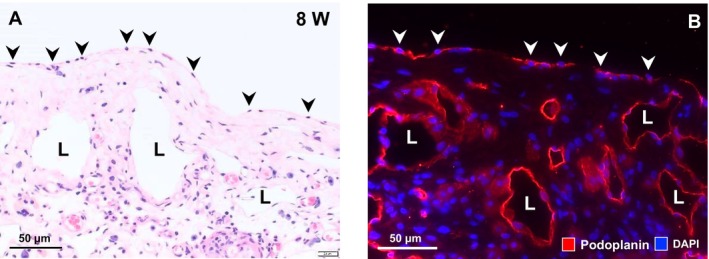
High‐magnification findings of the engrafted site at 8 weeks. (A) A single layer of flattened cells covers the pleura‐defective area (arrowheads). (B) Immunohistochemical findings. Single flat surface cells (arrowheads) and luminal structures (L) stained positive for podoplanin. Pleural mesothelial cells and lymphatic vessels were suggested, respectively. [Color figure can be viewed at wileyonlinelibrary.com]

At 8 and 12 weeks post‐transplantation, while the fibrous connective tissue remained (Figure 6E,F right), the engraftment of H‐APLTs proceeded to integration into the host pleura (Figure 6E,F left). At 8 weeks, dilated luminal structures developed in the connective tissue (Figure 6E right). Macrophages, plasma cells and neovascularization were observed (Figure 6e1). At 12 weeks, macrophages were observed around the luminal structures (Figure 6f1). The monolayer of flat cells appeared to be the pleural mesothelial cells, with the connective tissue covering the pleura‐defective area until 12 weeks post‐transplantation (Figure 6d2,e2,f2, PMC). High‐magnification images of the engrafted site at 8 weeks revealed a surface single flat cell layer immunostained for podoplanin, a marker of mesothelial cells (Figure 7A,B, arrowheads), demonstrating pleural regeneration at the defect site. Podoplanin is a well‐known marker of lymphatic vessel endothelial cells; luminal structures were positive for podoplanin in this study (Figure 7A,B,L).

**FIGURE 8:**
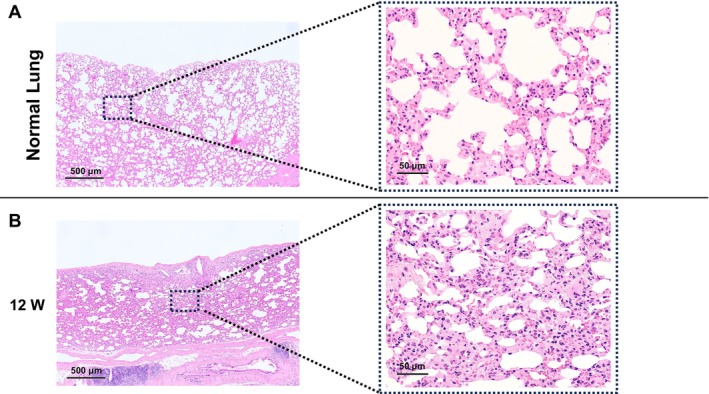
Lung parenchyma findings beneath the H‐APLT‐engrafted site. (A) The normal lung parenchyma and alveolar tissue. The right image is a magnification of the boxed area in the left image. (B) Lung parenchyma beneath the H‐APLT–engrafted site. The alveolar septa appeared to be thickened with fibrosis as compared with normal lungs. H‐APLT, hybrid artificial pleural tissue. [Color figure can be viewed at wileyonlinelibrary.com]

Lung parenchyma beneath the H‐APLT‐engrafted site was also observed (Figure 8B left), compared with the normal lung parenchyma (Figure 8A). Although the rats survived without respiratory symptoms 12 weeks post‐transplantation, a thickened alveolar septum was observed. In high‐magnification imaging, some fibrosis was observed in the alveolar septum, suggesting that the effects of inflammation following H‐APLT transplantation remained (Figure 8B, right).

## 4 Discussion

In this study, the hybrid biomaterial combined with a collagen‐coated PGA nanofiber sheet was generated and successfully implanted as an isograft. As transplantation of 3D layered cells is more effective than transplantation of a single layer of cells to mimic real biological tissue, pleural mesothelial cells and lung fibroblasts were co‐cultured. By adding accumulating FN‐G nanofilms coated fibroblasts, an artificial pleura was created with a more stable structure. The FN‐G nanofilms formed on the surfaces of lung fibroblasts provided interactive properties with the other cells, thereby promoting homogenous intercellular adhesion between pleural mesothelial cells and fibroblasts in a 3D structure. The FN‐G nanofilms would also induce cell survival, proliferation, signal transduction, and differentiation [21]. Previous study and our findings have suggested that fibroblasts may be effective in maintaining mesothelial cell integrity [24]. The accumulation of 3D‐layered fibroblasts reduced the expression of inflammatory cytokines and formed a satisfactory basal layer [22]. Formation of apical‐basal polarity, in other words, the location of mesothelial cells in the superficial layer and fibroblasts in the basal layer may have contributed to preserving their native properties and functions. In a study of peritoneal mesothelial cell transplantation, not only mesothelial cells on the surface of the artificial peritoneum but also submesothelial fibroblasts contributed to preventing intraperitoneal adhesions [32, 33]. Our results also suggest that fibroblasts and abundant ECM assist in maintaining mesothelial structure and function.

The survival of all rats without respiratory symptoms after H‐APLT transplantation in this study suggests that H‐APLT engraftment is an effective treatment in the rat air‐leak model. Particularly, the tissue with PGA nanofiber sheets assisted as a scaffold in maintaining tissue structure and mesothelial layer function. The preservation of mesothelial cells may have contributed to preventing intrathoracic adhesions, which are of concern with PGA sheets alone. We believe that the novelty of our research lies in the successful intrathoracic transplantation of a graft derived from mesothelial cells. By placing pleural mesothelial cells on the top layer, the H‐APLT becomes more physiological, allowing it to maintain the natural functions of mesothelium, such as inflammatory and immune responses, tissue repair, coagulation, and fibrinolysis [1, 2, 3, 4].

Despite no adhesion between the lungs and parietal pleura on macroscopic examination, inflammatory cell infiltration was observed post‐transplantation. The development of lymphatic vessels in the later stages is expected to be associated with these inflammatory conditions. Although PGA sheets are extensively used due to their ease of handling and convenience in affixation [7, 8, 9, 10, 11, 12, 13]; however, residual fibers can induce inflammatory reactions [13]. PGA shows less progressive degradation in acidic regions and accelerated degradation in alkaline regions [34]. It is hypothesized that in environments with lower pH as a result of inflammation, PGA degradation is slowed, leading to the retention of fibers. In vivo, as shown in Figure 6, remnants of PGA nanofibers were observed 2 weeks after transplantation. However, although the fibers were no longer visible after 4 weeks as indicated by the manufacturer's brochure, the inflammatory response remained. Porcine‐derived collagen and bovine‐derived fibronectin may also cause immunocyte infiltration due to heterogeneity of materials within the host. In the canine model, even 6 months after attaching the PGA nanofiber sheet, infiltration of inflammatory cells was observed [13]. Although the microscopic findings show that the inflammatory response and fibrosis do not resolve over time, it is unlikely to lead to postoperative complications because no intrathoracic adhesions were observed in the macroscopic findings.

This study has some limitations. First, it was not determined whether the covered mesothelium was derived from the graft or the host. Additionally, we were unable to devise a method to evaluate inflammatory reactions in vitro or assess the dynamic effects of inflammation on the lung.

Various challenges exist in the clinical application of pleural tissue engineering using mesothelial cells. First, the source of mesothelial cells has not yet been established. Although the greater omentum has been suggested as a source of mesothelial cells, additional operations under general anesthesia are necessitated for obtaining intact mesothelial cells for culture [35]. Effluent fluid from peritoneal dialysis patients has been investigated as a potential source of mesothelial cells, but is not available due to loss of mesothelial cell function [2, 35]. Mesothelial cells derived from dialysis effluent generally originate from uremic states and are prompted to differentiate into fibroblast‐like cells (mesothelial‐mesenchymal transition) [2, 35]. The quest for regeneration or a reliable source of mesothelial cells continues to drive research efforts. In a previous report, adipose‐derived stem cells were shown to promote the regeneration of damaged visceral pleural mesothelium [36]. Recently, the derivation of mesothelial progenitor‐like cells from induced pluripotent stem cells has reported potential for cell therapy [37]. In the future, differentiation and induction of mesothelial cells from human somatic stem cells may be achieved. Continued research and innovation hold promise for overcoming every issue and advancing pleural tissue engineering for the benefit of application in clinical practice.

## 5 Conclusion

Hybrid 3D tissue construction with mesothelial cells, fibroblasts, and PGA nanofiber sheets provided artificial pleural tissue with a highly organized mesothelium and structural stiffness in this study. The transplanted tissue engrafted well to prevent air leakage. Mesothelial function preservation may prevent intrathoracic adhesions. To our knowledge, this is the first study to utilize a hybrid 3D mesothelial tissue for a pleural defect model. However, further research investigating reducing the inflammatory response, securing sources of mesothelial cells, and achieving successful autologous transplantation is required. We hope that this study can contribute to the establishment of regenerative therapy of pleural mesothelial cells.

## Author Contributions

Kengo Tani, Daisuke Kimura, Yoshiya Asano, and Cheng‐Yang Song designed the study, analyzed the data and interpreted the results. Daisuke Kimura secured the funding from the Grants‐in‐Aid for Scientific Research (KAKENHI). Kengo Tani drafted the manuscript, while Daisuke Kimura, Yoshiya Asano, Cheng‐Yang Song, Hiroshi Shimoda, and Masahito Minakawa revised it. All authors have read and approved the final version of the manuscript.

## Conflicts of Interest

The authors declare no conflicts of interest.
